# Efficacy of Larval Therapy for Wounds: A Systematic Review and Meta-Analysis

**DOI:** 10.3390/jcm14020315

**Published:** 2025-01-07

**Authors:** Thao Lam, Gabriela E. Beraja, Hadar Lev-Tov

**Affiliations:** Dr. Phillip Frost Department of Dermatology and Cutaneous Surgery, Miller School of Medicine, University of Miami, Miami, FL 33136, USA

**Keywords:** larval therapy, maggot debridement therapy, wounds, ulcers, biosurgery

## Abstract

**Background/Objective**: Larval therapy (LT), an intervention using live fly larvae to remove necrotic tissue and promote healing, has regained attention in order to address the growing need for chronic wound management. LT was approved by the Food and Drug Administration (FDA) in 2004 to treat difficult-to-heal wounds; however, LT remains an underutilized therapy. To evaluate efficacy of LT in a systematic review and meta-analysis of wound outcomes from randomized controlled trials (RCTs). **Methods**: We followed the Preferred Reporting Items for Systematic Reviews and Meta-Analyses (PRISMA) guidelines to conduct a literature search across five databases for published and unpublished RCTs comparing LT to conventional therapy. A meta-analysis was performed to evaluate LT’s effect on debridement as the primary outcome. Wound healing, bioburden, and treatment-related pain were analyzed as secondary outcomes. Bias was assessed using Cochrane’s Risk-of-Bias 2 tool. **Results**: Eight RCTs were included in the review. The meta-analysis suggested that LT may be more effective for complete wound debridement (RR = 2.17), though this result was not significant (*p* = 0.09). The analysis is limited by the small number of studies and the high heterogeneity between studies (I^2^ = 75%). There were no significant differences in the healing rate, antimicrobial effects, or pain compared to conventional therapy. There is a moderate risk for bias in the selection of reported outcomes. **Conclusions**: LT is as effective as conventional therapy for debridement and may be an alternative for patients who cannot tolerate traditional methods. LT patients may experience similar levels of pain, but LT does not worsen wound healing or infection compared to those receiving routine care.

## 1. Introduction

Chronic wounds pose a significant global healthcare challenge, distinguished by their ongoing difficulty in healing as expected. These unhealed wounds not only lower quality of life but also raise healthcare costs and contribute to increased morbidity [1]. They affect individuals with diverse medical backgrounds and lifestyles, with causes ranging from vascular issues—such as venous, arterial, or mixed conditions—to diabetic foot ulcers and pressure ulcers [2].

The increasing prevalence of chronic leg ulcers, which impact 0.6% to 3% of people over the age of 60 and more than 5% of those over 80, is influenced by the aging population and risk factors for atherosclerotic occlusion, such as obesity, diabetes, and smoking [3,4,5]. These ulcers are a significant health concern, with prevalence rates in the general population ranging from 1.9% to 13.1% [3]. Approximately 1–2% of people in developing countries and almost 10% globally will develop a chronic wound at some point in their lives [4,6].

Chronic wound management is most effective when tailored to each person’s needs. The TIME (tissue, inflammation/infection, moisture imbalance, and epithelial edge advancement) concept describes a holistic approach to wound bed preparation (WBP), following a systematic method that facilitates wound healing [1,7]. The underlying causes of a wound, patient comorbidities, and the needs of a patient are considered to determine the appropriate course of action: whether a wound is likely to heal, needs ongoing maintenance, or is considered non-healable [8]. Successful WBP relies on debridement to eliminate necrotic tissue that can hinder healing. Debridement includes surgical, autolytic, chemical, mechanical, hydrosurgery, ultrasonic, enzymatic, and/or biologic methods [1].

Among the various methods used to support WBP, larval therapy (LT), or maggot debridement therapy, has re-emerged in recent decades as a distinctive and cost-effective form of biologic debridement for effective tissue removal and infection management. FDA-approved in 2004, LT utilizes the natural properties of newly hatched and sterilized larva from the green bottle fly (e.g., *Lucilia sericata* and *Lucilia cuprina*) to effectively separate a wound’s nonviable tissue from living tissue. It has gained recognition in managing difficult-to-heal wounds that have failed with conventional treatments and for addressing antibiotic resistance [9]. Clinical experience suggests that LT is effective in treating venous leg ulcers (VLUs), mixed leg ulcers (MLUs), diabetic foot ulcers (DFUs), burns, and many other skin conditions [9]. Despite this, LT remains heavily underutilized in clinical practice [10].

Recognizing the advantages of LT is essential for clinicians and caregivers striving to achieve optimal wound healing outcomes. Previous reviews of larval therapy include retrospective and uncontrolled studies, which may not account for potential confounders that under- or overestimate larval therapy’s effect on wounds [11,12]. This systematic review synthesizes the clinical efficacy of larval therapy, as demonstrated in randomized controlled trials (RCTs), by comparing direct/free-range larvae and indirect/larval bag therapy with conventional therapy (e.g., sharp debridement, wound dressings, or offloading) for wounds.

## 2. Materials and Methods

Protocol and search strategy: The study protocol followed the PRISMA guidelines [13] and was registered on PROSPERO (CRD42024576779). A systematic search for English and Spanish studies was performed on PubMed, Embase, CINAHL, and Scopus from their inception to July 2024. We used the medical subject headings “maggot debridement therapy” and “wounds and injuries” in our search to encompass as many studies as we could find. The Cochrane Central Register of Controlled Trials was also searched for gray literature and unpublished trials on larval therapy. References from included studies were also reviewed to search for additional studies. All studies were imported into Covidence systematic review software for the management of the review [14].

Inclusion and exclusion criteria: Two authors (T.L. and G.B.) independently screened abstracts of the imported studies for their suitability to be included. If there were differences, they would discuss these with each other, involving a third author (H.L.) if the disagreement could not be resolved. After title and abstract screening, the full texts of the remaining studies were reviewed to determine if they met the following selection criteria: (i) the study must be a published or unpublished randomized controlled trial (RCT), (ii) the control group must receive conventional or routine therapy, (iii) the treatment group receives live LT using any route (i.e., direct or indirect larval administration), and (iv) the study must involve only human subjects. The exclusion criteria included there being (i) data from review articles, editorials, case reports, or case series, (ii) in vitro or animal experiments, (iii) duplicate studies, (iv) data that cannot be accessed, and (v) control groups that do not align with conventional methods.

Data extraction: Two authors (T.L. and G.B.) independently extracted the following data from the included studies by using Covidence software (Veritas Health Innovation, Melbourne, Australia): first author, publication year, country, sample size, number of withdrawals, reason for withdrawals, treatment details, treatment duration, and outcomes, with wound debridement being the primary outcome. Secondary outcomes included complete wound healing, the eradication of *Staphylococcus aureus* and/or *Pseudomonas aeruginosa* cultures, and treatment-related pain scores. Discrepancies in data extraction were resolved via discussion between the two authors. The extracted data were exported into a Microsoft Excel file for review.

Quality assessment: Two authors (T.L. and G.B.) used the Cochrane risk-of-bias tool version 2.0 to evaluate the quality of the included studies with respect to the following domains: the randomization process, deviations from intended interventions, missing outcome data, measurement of the outcome, and selection of the reported result. The authors (T.L. and G.B.) also assessed the certainty of the evidence by using the grading of recommendation, assessment, development, and evaluation approach.

Statistical analysis: For continuous variables, the effect sizes were reported as standardized mean differences (SMDs) with 95% confidence intervals (CIs). A meta-analysis was performed on outcomes that had the respective data from at least three of the included studies by using a random effects model. Effect sizes for complete debridement, complete healing, and bacterial eradication were analyzed as dichotomous data and reported as risk ratios (RRs). If applicable, subgroup analyses by wound type and LT administration were also performed. The significance level was set at *p* < 0.05. Statistical heterogeneity was tested using Higgin’s and Thompson’s I^2^. I^2^ scores less than 25% were considered to show low heterogeneity, and scores above 50% indicate substantial heterogeneity.

## 3. Results

### 3.1. Study Selection

**Figure 1:**
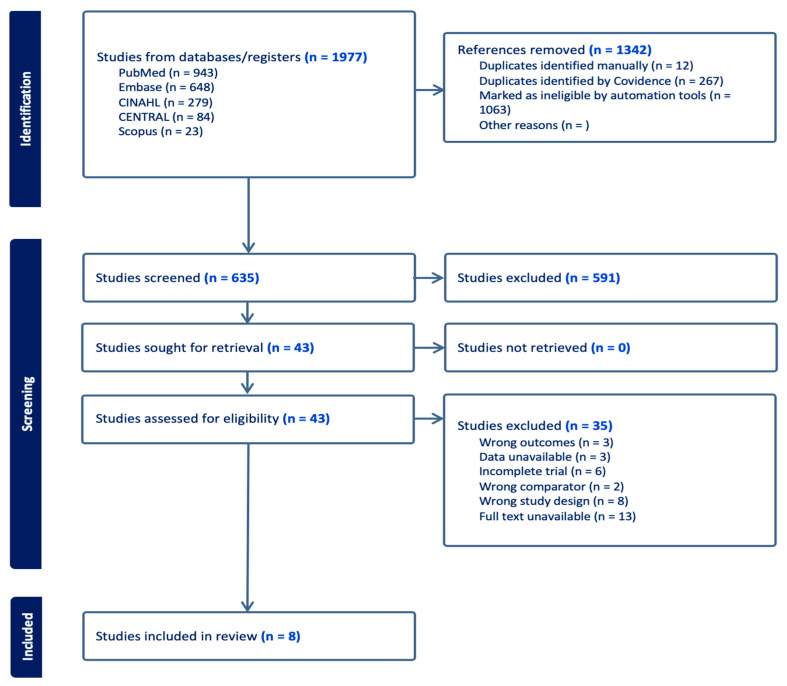
A flow diagram depicting the steps of the review following the PRISMA guidelines.

A total of 635 studies were identified from the databases and registers after removing duplicates. Those remaining were screened by their titles and abstracts to determine their suitability for the review, retrieving 43 articles for full-text screening against our inclusion and exclusion criteria. We excluded 35 studies, primarily due to the unavailability of full texts, incorrect study designs, or incomplete data (Figure 1). Eight eligible trials were included in the systematic review, including one unpublished RCT [15,16,17,18,19,20,21,22].

### 3.2. Study Characteristics

Eight studies were conducted across various countries: three in Iran [18,19,21], three in the UK [16,17,20], one in the United States [15], and one in France [22]. The sample sizes varied from 31 to 267 participants. The studies focused on certain types of wounds, including VLUs [15,16,17,20,22], MLUs [17,20], DFUs [15,19,21], pressure injuries [21], and full-thickness burns [18]. VLUs represented the largest portion of the sample size at 58%, followed by MLUs (19%) and DFUs (15%), with the remaining wound types accounting for less than 10%. The follow-up periods varied widely, ranging from 4 days up to 12 months [17,19].

Dumville et al. [17] used both loose and bagged larvae for their intervention group. In contrast, Mudge et al. [20], Opletalova et al. [22], and Cowan [15] only used bagged larvae, while Davies et al. [16] combined bagged larvae with a four-layer compression bandage. Gaffari et al. [18] were unique in their use of only loose larvae applied directly to the wounds. Nezakati et al. [21] and Malekian et al. [19] used loose larvae plus the standard of care, which included debridement, antibiotic therapy, and offloading, among other treatments.

All studies utilized *Lucilia sericata*, though the number of larvae and the application duration varied. Dumville et al. indicated that larvae were left on a wound for 3–4 days, while Cowan et al. applied the larvae every 4 days without specifying the quantity used. Among the studies that used bagged larvae, only Opletalova et al. reported the frequency of application, at twice a week with 80 larva per bag. Gaffari and Malekian et al. applied approximately five to ten larvae to every square centimeter of a wound. Mudge et al. and Davies et al. did not provide details regarding the frequency or quantity of larvae used in their treatment regimens.

Most studies compared their interventions to either hydrogel [17,20], routine care [18,19,21,22], or sharp debridement alone [15]. Table 1 outlines the specifics of the routine care used in each study, as there were slight variations among them. In contrast, Davies et al. only compared the intervention group to a four-layer compression bandaging group.

### 3.3. Complete Debridement

**Figure 2:**
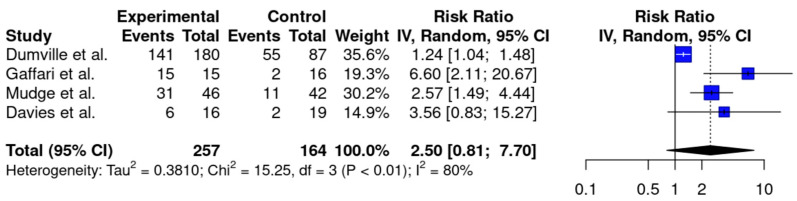
A forest plot with pooled meta-analysis data for the complete debridement of all wound types [16,17,18,20]. Vertical black line indicates a risk ratio (RR) of 1, indicating no difference between experimental and control groups. Blue squares indicate the RRs of the individual studies. Black diamond displays the 95% confidence interval of the meta-analysis. Vertical dashed line corresponds to the meta-analysis RR of 2.50.

Four RCTs [16,17,18,20] investigated debridement as an indicator of efficacy when comparing LT to conventional therapy, pooling a total of 421 patients. The largest trial comprised 267 participants, and the smallest included 31 [17,18]. Individually, most of these studies reported faster or improved debridement with LT; however, the meta-analysis of applicable studies showed no significant difference in complete debridement from LT compared to conventional therapy (RR = 2.50, CI: [0.81, 7.70], *p* = 0.09, I^2^ = 80%) (Figure 2). A subgroup analysis limited to studies on VLUs and MLUs [16,17,20,22] also showed no difference in debridement (RR = 1.88, CI: [0.52, 6.80], *p* = 0.17, I^2^ = 75%) (Supplementary Figure S1).

### 3.4. Secondary Outcomes

Four studies [16,17,18,21] assessed the efficacy of healing by comparing the proportion of healed wounds within specified time frames. Gaffari et al. and Nezakati et al. noted improved healing rates in the larval groups. Other studies did not note differences in wound healing with LT [16,17]. The meta-analysis demonstrated no significant differences in wound healing (RR = 1.33, CI [0.82, 2.18], *p* = 0.17, I^2^ = 77%) (Supplementary Figure S2).

Three RCTs [18,19,21] took bacterial cultures of wounds to observe changes in bioburden post-treatment. Two of these reported decreased infection of *S. aureus* and *P. aeruginosa* with larval treatment [19,21]. Using a random effects model, there were no differences in the eradication of *S. aureus* (RR = 1.51, CI [0.58, 3.96], *p* = 0.21, I^2^ = 44%) or *P. aeruginosa* (RR = 1.80, CI [0.29, 11.19], *p* = 0.30, I^2^ = 29%) (Supplementary Figures S3 and S4).

**Figure 3:**
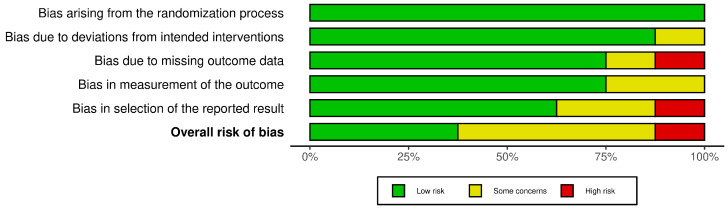
A summary of the bias risk for the included studies using version 2 of the Cochrane risk-of-bias tool for randomized trials.

Treatment-related pain was also evaluated in three trials [15,17,22] by utilizing visual analog scales (VASs), with higher scores indicating more severe pain. One trial reported significantly higher pain scores for groups with LT [17]. Cowan and Opletalova et al. noted no difference in pain scores between the two groups. On the meta-analysis, the standardized mean difference (SMD) was not significant (SMD = 0.51, CI [−0.42, 1.44], *p* = 0.18, I^2^ = 88%) (Supplementary Figure S5). Some studies took additional measures to assess the effect of pain on quality of life. Dumville et al. measured study participants’ perceptions of health-related quality of life (QOL) at the baseline and at three, six, nine, and twelve months. Mudge et al. asked Likert-type scale questions about participants’ experience with treatment, including comfort when wearing the dressings, comfort compared to previous treatments, and overall satisfaction. Cowan’s pain score assessments were part of a participant survey that also assessed satisfaction with the debridement method, the esthetic unpleasantness of the method, and the ease of care. No significant differences were found in these participant-reported measures between the larval therapy and control groups [15,17,20]. Assessments of risk of bias are demonstrated in Figure 3. Evaluation of certainty of the evidence is summarized in Table S2 in the supplementary material.

## 4. Discussion

We evaluated outcomes from LT by using high-level evidence from RCTs. The meta-analysis showed that none of these outcomes were significantly improved compared to conventional therapy, but the pooled data showed trends favoring improved outcomes from the larval groups in complete debridement, wound healing rates, and antimicrobial effects. Subgroup analyses on VLUs/MLUs showed similar results, suggesting that LT is as effective as—and possibly more effective than—conventional therapy for debridement.

The most-reported adverse effect of LT is wound pain, though the pooled analysis showed no significant difference in pain scores in LT cohorts compared to groups who had undergone the gold-standard sharp debridement. Treatment-related pain may be attributed to the removal of tissue by larvae’s mouth hooks and the sensation of larvae crawling across the wound surface. Thus, patient discomfort should be taken into consideration when conducting LT; however, wound-related pain is likely transient and does not significantly affect a patient’s experience after the intervention, since patient perceptions of the experience remain largely unchanged.

Larvae of *L. sericata* and related species feed on necrotic tissue as a source of nutrition [6]. They have mouth hooks for locomotion and to physically remove tissue from surfaces [23]. They also feed via external digestion, releasing excretions and secretions (E/S) containing proteolytic enzymes, deoxyribonucleases, and antimicrobial substances that facilitate the breakdown and liquification of tissue for consumption [24]. These processes make them ideal candidates for removing non-viable tissue from wounds, possessing both mechanical and enzymatic debridement properties [6]. Debridement prepares a wound bed to promote the growth of healthy granulation tissue. Therefore, LT may also result in improved wound healing overall. Chronic wounds experience an imbalance in their wound bed microbiomes, allowing pathogenic bacteria to flourish, particularly *S. aureus* and *P. aeruginosa* [19]. The antimicrobial effects of larval E/S may be helpful for disinfection and the removal of biofilms from wounds, especially with the current rise in antibiotic resistance [6]. Thus, wound infections are not a contraindication for LT, and LT may even be used as a palliative treatment. In patients with serious illness where the goals of care aim at optimizing quality of life and relieving suffering, LT has been used to reduce odor and infection for chronic and even fungating wounds [25,26].

**Figure 4:**
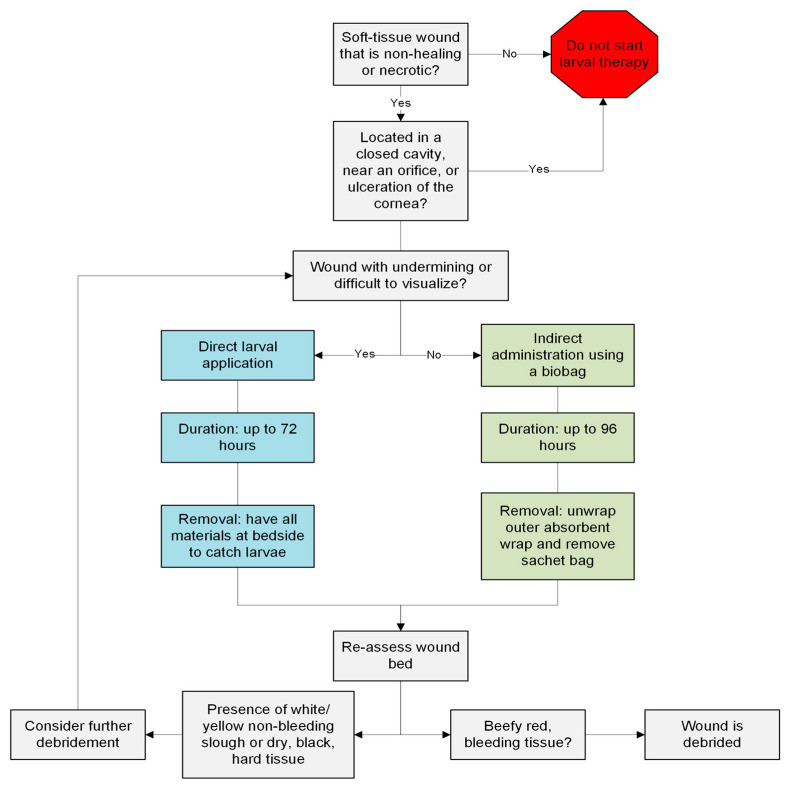
A guide for clinicians who are considering initiating larval therapy for wounds.

Many clinicians hesitate to initiate LT due to patients’ or their own disdain at the concept [10]; however, LT can be applied to a variety of wound types (Figure 4). In recognizing the potential benefits of LT and its restrictions, new innovations are being carried out to harness the enzymatic power of larval secretions while minimizing patient discomfort. The isolation of larval excretions and secretions into a recombinant proteolytic enzyme (RPE) has been shown to be a potential alternative [24]. A phase IIa clinical study of this RPE saw trends of concentration-dependent reductions in necrotic tissue and increases in wound bed granulation tissue [27]. Additionally, the RPE had an excellent side effect profile as there were minimal reports of pain at the wound sites during application or after prolonged contact with the wound bed over the treatment period [24,27]. This provides an added benefit in comparison to standard larval therapy or sharp debridement.

Limitations to this meta-analysis include high variation and potentially underpowered studies. Analyses included, at most, four RCTs for each outcome. Most outcomes had I^2^ scores over 50%, indicating high heterogeneity between studies. To be comprehensive, we included both direct and indirect larval administration at the expense of introducing variation. It is hypothesized that direct administration, which involves the introduction of free-range larvae directly into a wound, provides greater debridement since the larvae can move freely and reach areas that traditional dressings may not have access to. Indirect or bagged application is thought to be less efficient, as maggots are contained in biobag dressings and are not in direct contact with the wound bed [27].

Two studies [16,18] utilized computer-generated software for randomization, with one study specifically reporting the use of block randomization [17]. The remaining five studies indicated that randomization was performed but did not provide specific details about the sequence generation. Without transparency regarding the randomization details, it is difficult to assess the reliability of their methods, possibly introducing bias that could affect study outcomes. Additionally, two studies reported specifically stratifying participants based on wound size or area [17,20]. Two studies blinded only the assessors [17,20], and two other studies successfully blinded both the participants and assessors [21,22]. The rest did not implement blinding for either the participants or the assessors. Notably, Opletalova et al. ensured that participants were unaware of their treatment group by requiring them to wear blindfolds during dressing changes, making it the most robust blinding method among all of the studies.

Half of the studies reported adverse effects during the trial period [15,16,17,22]. Some reported serious adverse events unrelated to the interventions (e.g., leg bone osteitis, death, and acute urinary retention) [17,22]. Overall, some studies provided insights into adverse effects while others did not. This reporting variability suggests bias in the selection of the reported results (Figure 3). There is a need for standardized protocols to ensure that all relevant side effects are consistently documented and analyzed, which would enhance the overall understanding of the interventions’ safety and efficacy.

Gaffari et al. included only male participants and had a relatively small sample size (n = 31), raising concerns about the generalizability and representativeness of their findings. Lastly, Opletalova et al. demonstrated a high level of transparency regarding attrition, as the table notes provided information about missing data. Conversely, Davies et al. initially indicated that there were 40 total participants, but failed to provide further details on attrition, as we were only able to extract data from 35 participants. This discrepancy suggests a lack of clarity in Davies et al.’s reporting. and raises questions about missing outcome data. Such inconsistencies can lead to confusion regarding the study’s sample size and potentially affect the reliability of the results. It underscores the importance of accurate reporting and transparency in clinical trials to ensure that the findings can be properly interpreted.

## 5. Conclusions

Despite LT’s low utilization in clinical practice, the meta-analysis suggests that LT is an effective debridement agent and may be used as an alternative to standard sharp surgical debridement in frail patients who cannot tolerate surgical debridement, or those with dry, necrotic, or infected wounds; however, it is difficult to generalize these findings given the scarcity of RCTs available and the high heterogeneity between these studies. Current research to isolate the proteolytic properties of larval E/S can introduce alternative devices that may perform as well as LT or conventional therapy.

## Figures and Tables

**Table 1:** Summary characteristics of the RCTs included.

Author Year	Country	Wound Type	Mean Age (Years)	Total (N)	Experimental (n)	Control (n)	Larval Application	Outcomes	Follow-Up Duration
Dumville et al., 2009 [17]	United Kingdom	VLU and MLU	74	267	Direct LT (94), indirect LT (86)	Hydrogel (87)	Left on for 3–4 days	a, b, c, d, and e	6 to 12 months
Gaffari et al., 2023 [18]	Iran	FTB	48	31	Direct LT (15)	Routine—SD, silver dressings, antibiotics, and offloading (16)	3–4 times/week (5–10 larvae/cm^2^)	a, b, c, d, and f	6 days
Mudge et al., 2014 [20]	United Kingdom	VLU and MLU	72	88	Indirect LT (46)	Hydrogel (42)	N/A	a and b	28 to 35 days
Davies et al., 2015 [16]	United Kingdom	VLU	77	40	Indirect LT + 4LB (20)	4LB (20)	N/A	a, c, and e	12 weeks
Nezakati et al., 2020 [21]	Iran	DFU and Bedsore	55	90	Direct LT + routine (45)	Routine— SD, wet dressing, nutrition support, and antibiotics (45)	2 times/week (8–10 larvae/cm^2^)	c and d	3 weeks
Malekian et al., 2019 [19]	Iran	DFU	61	50	Direct LT + routine (25)	Routine— antibiotics, SD, and offloading (25)	Every 2 days (5–7 larvae/cm^2^)	d	4 days
Opletalova et al., [22] 2012	France	VLU	73	119	Indirect LT (58)	Routine—SD, hydrogel/hydrocolloid or alginate/fiber-based (61)	2 times/week (80 larvae/bag)	b, d, e, and g	8, 15, and 30 days
Cowan 2014 [15]	United States	VLU, DFU, and other	65	45	Indirect LT (23)	SD (22)	Every 4 days	b and e	8 days

VLU (venous leg ulcer), MLU (mixed leg ulcer), FTB (full-thickness burn), DFU (diabetic foot ulcer), LT (larval therapy), SD (sharp debridement), and 4LB (four-layer compression bandage). a = time to debridement, b = treatment-related pain, c = time to healing, d = bacterial growth, e = adverse events, f = % slough, and g = % healing.

## Data Availability

The original contributions presented in this study are included in the article. Raw data supporting the conclusions of this article will be made available by the authors on request. Inquiries can be directed to the corresponding author.

## Supplementary Materials

The following supporting information can be downloaded at: https://www.mdpi.com/article/10.3390/jcm14020315/s1, Figure S1. Forest plot for complete debridement of venous and mixed leg ulcers; Figure S2. Forest plot for wound healing; Figure S3. Forest plot for *Staphylococcus aureus* cultures; Figure S4. Forest plot for *Pseudomonas aeruginosa* cultures; Figure S5. Forest plot for treatment-related pain; Table S1. Excluded studies and reasons for exclusion; Table S2: GRADE Summary of Findings Table.
