# Supplementary material for: Efficacy of Larval Therapy for Wounds: A Systematic Review and Meta-Analysis
Source: J Clin Med. 2025 Jan 7;14(2):315. doi: 10.3390/jcm14020315 (PMC11765813; doi:10.3390/jcm14020315)
Supplement: Supplementary file 1 [file jcm-14-00315-s001.zip › jcm-3392773-supplementary.pdf]

Supplementary Material

PICO Methodology

Population/Patient: Adults with acute and chronic wounds
Intervention: Larval therapy (i.e., direct/free-range and indirect/larval bag)
Comparison: Conventional therapy (e.g., sharp debridement, wound dressings, or offloading)
Outcome: Improvement in time-to-debridement, wound healing rate, bacterial burden, and ulcer-related pain

PICO Question: Among adults with acute and chronic wounds, do RCTs comparing larval therapy (i.e., direct/free-range and indirect/larval bag) versus conventional therapy (e.g., sharp debridement, wound dressings, or offloading) demonstrate differences in time-to-debridement, rate of wound healing, reduction in bacterial burden, and ulcer-related pain across diverse global populations?

Search Strategies:

PubMed:
(("Debridement"[Mesh] AND "Larva"[Mesh]) OR "Maggot Debridement Therapy"[Mesh] OR ((Maggot*[tiab] or larva*[tiab]) AND (debrid*[tiab] or therap*[tiab] or treat*[tiab]))) AND ("Wounds and Injuries"[Mesh] OR burn*[tiab] OR ulce*[tiab] OR diabet*[tiab] OR venous*[tiab])

Cochrane:
#1 (maggot* OR larva*):ti,ab,kw AND (debrid* OR therap* OR treat*):ti,ab,kw
#2 MeSH descriptor: [Wounds and Injuries]
#3 MeSH descriptor: [Debridement]
#4 MeSH descriptor: [Larva]
#5 MeSH descriptor: [Maggot Debridement Therapy]
#6 ((#3 AND #4) OR #5 OR #1) AND (#2 OR (burn* OR ulce* OR diabet* OR venou*):ti,ab,kw)

Scopus:
(ALL (("Debridement" [mesh] AND "Larva" [mesh]) OR "Maggot Debridement Therapy" [mesh]) AND ALL ("Wounds and Injuries" [mesh]) OR TITLE-ABS-KEY (burn* OR ulce* OR diabet* OR venous*))

CINAHL:
((MH "Debridement+") AND (MH "Larval Therapy")) AND (((MH "Wounds and Injuries+") OR TI (burn* OR ulce* OR diabet* OR venous*) OR AB (burn* OR ulce* OR diabet* OR venous*)))

Embase:
('maggot therapy'/de OR maggot*:ti,ab,kw OR larva*:ti,ab,kw) AND (debride*:ti,ab,kw OR therap*:ti,ab,kw) AND ('injury'/exp OR wound*:ti,ab,kw OR injur*:ti,ab,kw OR burn*:ti,ab,kw OR diabet*:ti,ab,kw OR venous*:ti,ab,kw) NOT ('animal'/exp NOT 'human'/exp OR 'case report'/de)

Secondary Analyses Forest Plots

Figure S1. Forest plot for **Complete debridement of venous and mixed leg ulcers**

	Experimental		Control			Risk Ratio
**Study**	**Events**	**Total**	**Events**	**Total**	**Weight**	**IV, Random, 95% CI**
Dumville et al.	141	180	55	87	49.1%	1.24 [1.04; 1.48]
Mudge et al.	31	46	11	42	37.1%	2.57 [1.49; 4.44]
Davies et al.	6	16	2	19	13.9%	3.56 [0.83; 15.27]
**Total (95% CI)**		**242**		**148**	**100.0%**	**1.88 [0.52; 6.80]**

Heterogeneity: $Tau^2 = 0.2057$; $Chi^2 = 7.96$, df = 2 (P = 0.02); $I^2 = 75\%$

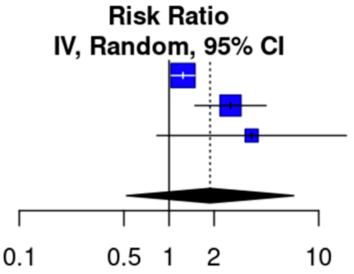

Figure S2. Forest plot for **Wound healing**

	Experimental		Control			Risk Ratio
**Study**	**Events**	**Total**	**Events**	**Total**	**Weight**	**IV, Random, 95% CI**
Dumville et al.	91	180	38	87	26.9%	1.16 [0.88; 1.53]
Gaffari et al.	15	15	16	16	32.6%	1.00 [0.88; 1.13]
Davies et al.	17	20	10	20	19.1%	1.70 [1.06; 2.73]
Nezakati et al.	33	45	17	45	21.4%	1.94 [1.28; 2.94]
**Total (95% CI)**		**260**		**168**	**100.0%**	**1.33 [0.81; 2.18]**

Heterogeneity: $Tau^2 = 0.0736$; $Chi^2 = 12.81$, df = 3 (P < 0.01); $I^2 = 77\%$

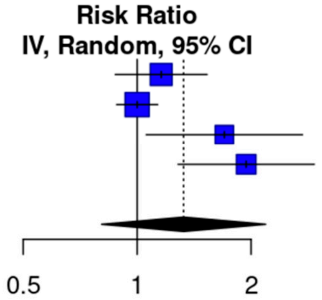

Figure S3. Forest plot for ***Staphylococcus aureus* cultures**

	Experimental		Control			Risk Ratio
**Study**	**Events**	**Total**	**Events**	**Total**	**Weight**	**IV, Random, 95% CI**
Gaffari et al.	7	7	5	8	44.1%	1.55 [0.94; 2.54]
Nezakati et al.	22	33	14	29	54.4%	1.38 [0.88; 2.16]
Malekian et al.	9	9	0	9	1.5%	19.00 [1.28; 282.12]
**Total (95% CI)**		**49**		**46**	**100.0%**	**1.51 [0.58; 3.96]**

Heterogeneity: $Tau^2 < 0.0001$; $Chi^2 = 3.55$, df = 2 (P = 0.17); $I^2 = 44\%$

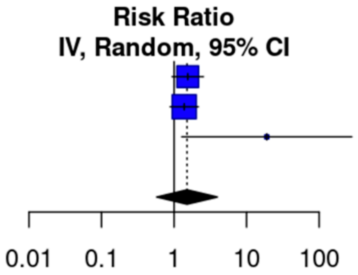

Figure S4. Forest plot for ***Pseudomonas aeruginosa* cultures**

	Experimental		Control			Risk Ratio
**Study**	**Events**	**Total**	**Events**	**Total**	**Weight**	**IV, Random, 95% CI**
Gaffari et al.	2	7	3	10	21.6%	0.95 [0.21; 4.29]
Nezakati	1	1	2	4	71.6%	1.80 [0.79; 4.11]
Malekian	8	9	0	7	6.8%	13.42 [0.91; 197.06]
**Total (95% CI)**		**17**		**21**	**100.0%**	**1.80 [0.29; 11.19]**

Heterogeneity: $Tau^2 < 0.0001$; $Chi^2 = 2.83$, df = 2 (P = 0.24); $I^2 = 29\%$

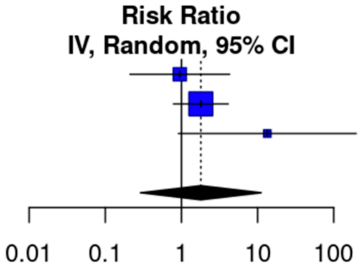

Figure S5. Forest plot for **Treatment-related pain**

Study	Experimental Mean	Experimental SD	Experimental Total	Control Mean	Control SD	Control Total	Weight	Std. Mean Difference IV, Random, 95% CI
Dumville et al. (Direct)	88.30	47.2000	82	41.80	43.9000	73	26.8%	1.01 [0.68; 1.35]
Dumville et al. (Indirect)	86.20	51.3000	73	41.80	43.9000	73	26.7%	0.93 [0.58; 1.27]
Opletova et al.	2.30	2.6000	51	2.70	2.6000	54	26.1%	-0.15 [-0.54; 0.23]
Cowan	4.14	3.1100	14	3.63	3.2840	16	20.3%	0.15 [-0.56; 0.87]
**Total (95% CI)**			**220**			**216**	**100.0%**	**0.51 [-0.42; 1.44]**

Heterogeneity: $Tau^2 = 0.2984$; $Chi^2 = 25.56$, df = 3 ($P < 0.01$); $I^2 = 88\%$

Std. Mean Difference
IV, Random, 95% CI

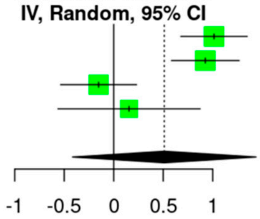

Table S1. Excluded studies and reasons for exclusion

Citation	Reason for exclusion
Abernethy, AP. Palliative Care Pharmacotherapy Literature Summaries and Analyses. Journal of Pain & Palliative Care Pharmacotherapy. Mar 2010;24(1):56–61.	Study design
Abstracts of the 48th EASD Annual Meeting of the European Association for the Study of Diabetes. Diabetologia 55. 2012;(Suppl 1):1–538.	Cannot access data
ASL 2. Multicenter, randomised, open-label, parallel group, controlled study to evaluate the efficacy, tollerability and safety of BioFOAM dressing containing larve of Lucilia sericata in patients with chronic, vascular ulcers with/without evident infection versus standard treatment. EudraCT Identifier: 2008-006076-30. Posted Apr 20, 2009. Accessed July 30, 2024. https://www.clinicaltrialsregister.eu/ctr-search/trial/2008-006076-30/IT	Incomplete study
Bolton LL. Evidence corner. Debriding pressure ulcers with maggot versus conventional therapy. Wounds: A Compendium of Clinical Research & Practice ;18(9):A19-A22.	Cannot access data
Boon, H, Freeman, L, Unsworth, J. Wound care. Larvae help debridement. Nurs Times. 1996 Nov;92(46):76-8, 80.	Cannot access data
Brin YS, Mumcuoglu KY, Massarwe S, Wigelman M, Gross E, Nyska M. Chronic foot ulcer management using maggot debridement and topical negative pressure therapy. J Wound Care. 2007 Mar;16(3):111-3.	Study design
Cambal M, Labas P, Kozanek M, Takac P, Krumpalova Z. Maggot debridement therapy. Bratisl Lek Listy. 2006;107(11-12):442-4.	Study design
Cangel U, Sirekbasan S, Polat E. Comparison of Larval Therapy and Vacuum-Assisted Closure Therapy after Revascularization in Peripheral Artery Disease Patients with Ischemic Wounds. Evid Based Complement Alternat Med. 2022 Mar 29;2022:8148298.	Study design
Dehghan O, Tabaie SM, Rafinejad J, Toutounchi M, Tiyuri A, Azarmi S, Esmaeeli Djavid G, Akbarzadeh K. A Parallel Randomized Clinical Trial for Comparison of Two Methods of Maggot Therapy, Free-Range Larvae and Larval-bag, in Diabetic Ulcer (Wagner 2). Int J Low Extrem Wounds. 2024;23(1):133-139.	Study design
Evans P. Larvae therapy and venous leg ulcers: reducing the 'yuk factor'. J Wound Care. 2002;11(10):407-8.	Study design
Igata, T. The usefulness and development of maggots therapy. UMIN Clinical Trials Registry Identifier: UMIN000024012. Posted Mar 24, 2014. Accessed July 30, 2024.	Incomplete study
Igawa, S. Maggot therapy using medical aseptic flies larvae for refractory chronic skin ulcer. UMIN Clinical Trials Registry Identifier: UMIN000018861. Updated Sep 4, 2021. Accessed July 30, 2024.	Study design
Jafari A, Hosseini SV, Hemmat HJ, Khazraei H. *Lucillia Sericata* larval therapy in the treatment of diabetic chronic wounds. J Diabetes Metab Disord. 2022 Jan 27;21(1):305-312.	Study design
Kerman Medical University. Maggot Therapy in Diabetic Foot Ulcers. ClinicalTrials.gov Identifier: NCT01681160. Updated Sep 10, 2012. Accessed July 30, 2024. https://clinicaltrials.gov/study/NCT01681160	Cannot access data
Maggot 2006 Ltd. A RANDOMIZED, OPEN, SELF-CONTROLLED CLINICAL TRIAL TO ASSESS THE SAFETY AND EFFICACY OF LARVAL THERAPY IN PATIENTS WITH LEG ULCERS. EudraCT Identifier: 2011-001284-44. Posted Apr 15, 2011. Accessed July 30, 2024. https://www.clinicaltrialsregister.eu/ctr-search/trial/2011-001284-44/HU	Incomplete study
Maggots can heal diabetic wounds. Podiatry Now. Dec 2011;14(12):8-8.	Cannot access data

Maggot therapy shown to be similar to standard leg ulcer care. Podiatry Now. Jul 2009;12(7):1p-1p.	Study design
Markevich, YO, McLeod-Roberts, J, Mousley, Melloy, E. Maggot therapy for diabetic neuropathic foot wounds. Diabetologia. 2000;43(Suppl 1): A15	Cannot access data
Martirosyan, EV. Investigation of antibacterial properties of sterile medical maggots in conditions of their application to deep burn wounds. New Armenian Medical Journal 2014;8(4):33-39.	Study design
Miyamoto, M. Assessment of maggot debridement therapy for leg ulcer. UMIN Clinical Trials Registry Identifier: UMIN000006165. Posted Aug 15, 2011. Accessed July 30, 2024.	Study design
Miyanaga, T. Evaluation of medical sterile maggot therapy on intractable gangrene and ulcer. UMIN Clinical Trials Registry Identifier: UMIN000011439. Updated Sep 29, 2022. Accessed July 30, 2024.	Cannot access data
Nonhealing ulcers respond best to maggot therapy. Home Healthcare Nurse. Sep 2003;21(9):577-577.	Cannot access data
Saghafipour, A, Akbarzadeh, K. Comparison of the effectiveness of maggot therapy in removing necrotic tissues and pathogenic bacteria in diabetic foot ulcers in grade of Wagner 2. International Clinical Trials Registry Identifier: IRCT20170723035248N2. Posted Mar 21, 2018. Accessed July 30, 2024.	Cannot access data
Schouten, H, Knippels, MCJ, Franken, RJPM. Fly maggots in the wound: Debridement, disinfection and wound healing. Nederlands Tijdschrift voor Geneeskunde. 2009;153(41):2022-2024.	Cannot access data
Soares MO, Iglesias CP, Bland JM, Cullum N, Dumville JC, Nelson EA, Torgerson DJ, Worthy G; VenUS II team. Cost effectiveness analysis of larval therapy for leg ulcers. BMJ. Mar 2009;338:b825.	Study design
Suarez L. Maggot therapy an effective treatment for open wounds. Orthopedics Today. Sep 2004;24(9):76-80.	Cannot access data
The First Affiliated Hospital of Dalian Medical University. Maggot Debridement Therapy Versus Conventional Dressing Therapy to Treat Diabetic Foot Ulcers (MDTDF). ClinicalTrials.gov Identifier: NCT02816749. Updated July 1, 2016. Accessed July 30, 2024.	Incomplete study
Thomas S, Jones M. Maggots can benefit patients with MRSA. Practice Nurse ;20(2):101-104	Cannot access data
University of Manchester. The Potential use of Larval Therapy/Biogun and Silver to Reduce Colonisation of MRSA in Diabetic Foot Ulcers. EudraCT Identifier: 2007-002571-14. Posted Mar 6, 2007. Accessed July 30, 2024. https://www.clinicaltrialsregister.eu/ctr-search/trial/2007-002571-14/GB	Study design
Wang, A. Clinical trial of Maggot debridement in the treatment of diabetic foot wounds. Chinese Clinical Trial Registry Identifier: ChiCTR2200056365. Posted Feb 4, 2022. Accessed July 30, 2024.	Incomplete study
Wayman J, Nirojogi V, Walker A, Sowinski A, Walker MA. The cost effectiveness of larval therapy in venous ulcers. J Tissue Viability. 2000 Jul;10(3):91-4. doi: 10.1016/s0965-206x(00)80036-4. Erratum in: J Tissue Viability 2001 Jan;11(1):51.	Study design
Wayman, J, Walker, A, Sowinski, A, Walker, MA. Larval debridement therapy: a cost-effective alternative to hydrogel in necrotic venous ulcers: a randomized trial. British Journal of Surgery 2000;87(4):507.	Study design
Young, T. Maggot therapy in wound management. Community Nurse. 1997; 3(8):43-45.	Cannot access data
ZooBiotic Limited. Clinical Trial of Maggots for Cleaning Leg Ulcers. EudraCT Identifier: 2007-005775-34. Posted Nov 26, 2007. Accessed July 30, 2024. https://www.clinicaltrialsregister.eu/ctr-search/trial/2007-005775-34/GB	Incomplete study

Table S2: GRADE Summary of Findings Table

**Larval therapy compared with conventional wound care**			
**Population/Patient:** Adults with acute and chronic wounds<br>**Setting:** Outpatient or inpatient setting<br>**Intervention:** Larval therapy (direct/free-range and indirect/larval bag)<br>**Comparison:** Conventional therapy (e.g., sharp debridement, wound dressings, or offloading)			
**Outcomes**	**No of Participants (studies)**	**Quality of the evidence (GRADE)**	**Relative effect (95% CI)**
Complete debridement	421<br>(4 RCTs)<br>[16-18,20]	⊕⊕⊝⊝<br>**Low**[1,2,4]<br>due to inconsistency and imprecision	RR 2.50<br>(0.81, 7.70)
Wound healing	428<br>(4 RCTs)<br>[16-18,21]	⊕⊕⊝⊝<br>**Low**[1,2,4]<br>due to inconsistency and imprecision	RR 1.33<br>(0.82, 2.18)
Wound-related pain (VAS pain scale from 0, no pain, to 10, maximum pain)	436<br>(3 RCTs)<br>[15,17,22]	⊕⊕⊝⊝<br>**Low**[1,2,4]<br>due to inconsistency and imprecision	SMD 0.51<br>(-0.42, 1.44)
Bacterial burden (*S. aureus* and *P. aeruginosa* cultures)	133<br>(3 RCTs)<br>[18,19,21]	⊕⊕⊕⊝<br>**Moderate**[1],[3]<br>due to imprecision	RR 1.56 (0.99, 2.44)
RCT: Randomized controlled trial, **RR**: Risk ratio; **CI**: Confidence interval			
GRADE Working Group grades of evidence<br>**High quality**: Further research is unlikely to change our confidence in the estimate of effect.<br>**Moderate quality**: Further research is likely to have an important impact on our confidence in the estimate of effect and may change the estimate.<br>**Low quality**: Further research is very likely to have an important impact on our confidence in the estimate of effect and may change the estimate.<br>**Very low quality**: We are very uncertain about the estimate.			
[1]All studies were randomized and blinding of outcomes were attempted to some degree in majority of the studies. We did not downgrade because the overall risk of bias was felt to be low.<br>[2]Due to high heterogeneity among the studies included in assessment of debridement, wound healing, and wound-related pain (I^2>70%), we decided to downgrade by one level for inconsistency.<br>[3]In assessing for positive cultures of bacteria, $I^2 = 24\%$. We did not downgrade because the inconsistency was felt to be low for this outcome.<br>[4]Given the small sample sizes, the optimal information size criterion is not met, so we decided to downgrade by one level for imprecision.			
